# *Salmonella* Serotypes Associated with Illnesses after Thanksgiving Holiday, United States, 1998–2018

**DOI:** 10.3201/eid2801.211986

**Published:** 2022-01

**Authors:** Farrell A. Tobolowsky, Zhaohui Cui, Robert M. Hoekstra, Beau B. Bruce

**Affiliations:** Centers for Disease Control and Prevention, Atlanta, Georgia, USA

**Keywords:** Salmonella, Salmonella infection, holidays, food, turkeys, crossover studies, serogroup, Thanksgiving, United States, bacteria, foodborne illness, enteric infections, food safety

## Abstract

We sought to determine which *Salmonella* serotypes cause illness related to the Thanksgiving holiday in the United States and to foods disproportionately eaten then (e.g., turkey). Using routine surveillance for 1998–2018 and a case-crossover design, we found serotype Reading to be most strongly associated with Thanksgiving.

Thanksgiving, celebrated annually in the United States on the fourth Thursday of November, often brings together family and friends who eat specific traditional foods, such as mashed potatoes, cranberry sauce, and pumpkin pie; the most prominent food eaten is turkey (*1*). In 2017, ≈45 million turkeys were produced for Thanksgiving, ≈18% of annual production (*2*). Turkey is popular across regions, races, sexes, and generations; 88% of persons in the United States report eating turkey during their Thanksgiving meal (*1*,*3*,*4*).

Foodborne *Salmonella* infections cause substantial illness and death in the United States: an estimated 1 million cases, 20,000 hospitalizations, and 400 deaths occur annually (*5*). Typical illness consists of diarrhea, fever, and abdominal pain lasting 3–7 days; only a minority of persons seek health care. Incubation typically ranges from 6 hours to 6 days (*5*). *Salmonella* outbreaks caused by serotypes Hadar and Saint Paul have been most commonly attributed to turkey, and serotypes Enteritidis, Heidelberg, and Typhimurium have been frequent causes of turkey-associated outbreaks (*6*). During 2015‒2020, Reading and Hadar were the serotypes most often isolated from turkeys (*7*); less is known about which serotypes cause turkey-associated sporadic *Salmonella* infections. We aimed to determine which *Salmonella* serotypes cause sporadic enteric infections after the Thanksgiving holiday and are most likely related to foods disproportionately eaten then, particularly turkey.

## The Study

The Laboratory-based Enteric Disease Surveillance (LEDS) system captures enteric infections with *Salmonella* species in the United States through passive surveillance of laboratory-confirmed isolates. State and territorial public health laboratories serotype *Salmonella* isolates; any unusual serotypes are sent to the Centers for Disease Control and Prevention’s National *Salmonella* Reference Laboratory (National Center for Emerging and Zoonotic Infectious Diseases, Division of Foodborne, Waterborne, and Environmental Diseases, Enteric Diseases Laboratory Branch) for further characterization. This surveillance system obtains demographic information; specimen source; collection date; test result; serotype; and, if available, outbreak association. We included infections that had fully serotyped *Salmonella* isolates that occurred from 1998 through 2018 and excluded isolates confirmed to be outbreak-associated.

Using a case-crossover design, we determined a case window for each year using the date of Thanksgiving, a minimum incubation period, and a window length. To account for seasonal variation in infections, we created nonexposure case windows before and after Thanksgiving by using the same case window length with a washout period separating the exposure and nonexposure windows (Appendix Figure). Our primary analysis used a 7-day case window after a minimum 2-day incubation period after Thanksgiving, with a 5-day washout. Control windows were also 7 days.

We conducted sensitivity analyses evaluating different minimum incubations, case windows, and washouts and stratified all analyses by serotype. To account for reporting biases in *Salmonella* cases during holidays and nonholidays, we used all *Salmonella* cases associated with serotypes other than the serotype under consideration as the comparison group. We calculated odds ratios (ORs) for the entire study period and compared ORs for 1998–2007 with those for 2008–2018. We conducted descriptive demographic and clinical analyses with SAS versions 9.4 (https://www.sas.com) and calculated ORs and unadjusted p values with R version 3.6.1 (R Foundation for Statistical Computing, https://www.r-project.org) using unconditional maximum-likelihood estimation with 95% CIs from the normal approximation.

Among 846,449 patients reported during 1998–2018 (Appendix Table 1), median patient age was 27 years; 52.5% of patients were female, and 82.2% were white. Isolates were identified by culture (n = 823,793 [99.8%]) and culture-independent diagnostic testing (n = 1,919 [0.2%]). Specimens were most commonly obtained from stool (86.8%), urine (6.3%), and blood (6.3%). The most frequent serotypes were Enteritidis (142,687 [18.3%]), Typhimurium (131,216 [16.8%]), and Newport (82,155 [10.5%]).

In our primary analysis, serotype Reading had the highest OR of association with the Thanksgiving holiday (2.18, 95% CI 1.58–3.01; p<0.0001) (Figure). Other serotypes with significantly increased ORs were Baildon (OR 1.92, 95% CI 1.04–3.52; p = 0.03), Worthington (OR 1.87, 95% CI 1.06–3.3; p = 0.03), Ohio (OR 1.74, 95% CI 1.06–2.86; p = 0.03), Hadar (OR 1.66, 95% CI 1.35–2.05; p<0.0001), Derby (OR 1.57, 95% CI 1.2–2.06; p = 0.001), Brandenburg (OR 1.42, 95% CI 1.01–2.01; p = 0.045), Schwarzengrund (OR 1.40, 95% CI 1.06–1.86; p = 0.02), 4,[5],12:i- (OR 1.39, 95% CI 1.25–1.54; p<0.0001), and Heidelberg (OR 1.34, 95% CI 1.21–1.49; p<0.0001). We found no other significant positive associations among serotypes with >50 patients who became ill during the 2–9 days after Thanksgiving.

**Figure:**
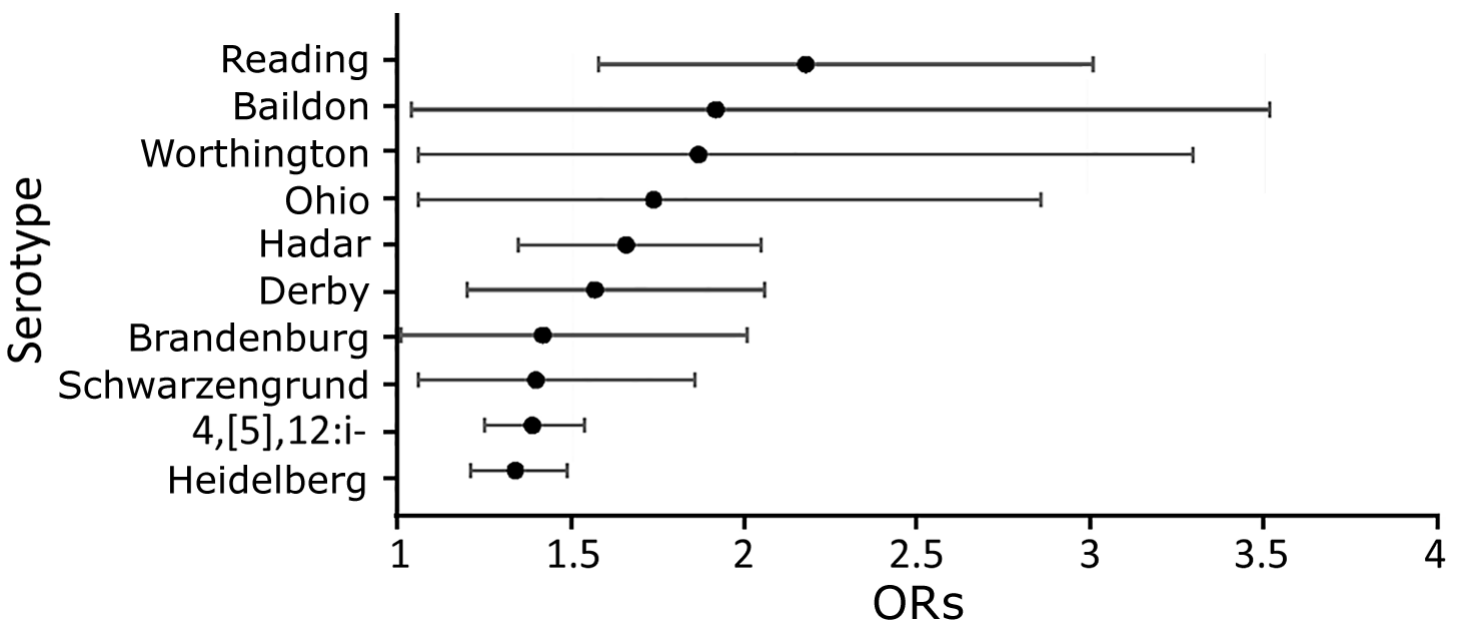
ORs for *Salmonella* serotypes associated with the Thanksgiving holiday, United States, 1998‒2018. Error bars indicate 95% CIs. No significant positive associations with Thanksgiving were found among other serotypes with >50 patients who became ill during 2–9 days after Thanksgiving (i.e., Litchfield, Braenderup, Infantis, Senftenberg, Anatum, Dublin, Mbandaka, Typhimurium, and Javiana.) Serotypes with >50 patients inversely associated with Thanksgiving and significant p values include Poona (OR 0.77), Panama (OR 0.71), Newport (OR 0.93), and Paratyphi B var. L(+) tartrate+ (OR 0.75). These serotypes probably are associated with foods not eaten more frequently on Thanksgiving or other exposures not more frequently experienced on Thanksgiving than other times of the year. OR, odds ratio.

In sensitivity analyses, case windows with varying durations of minimum incubation (range 0–12 days), illness (range 5–7 days), and washout (range 0–5 days) produced similar sets of serotypes with significantly increased ORs, most commonly Reading, Hadar, 4,[5],12:i-, Derby, Heidelberg, and Schwarzengrund (Appendix Table 2). Comparing 1998–2007 with 2008–2018, ORs for Baildon, Derby, Hadar, 4,[5],12:i:-, and Ohio increased by >45% from the first to the second period, but significantly so only for 4,[5], 12:i:-. Three more serotypes had significant ORs in only 1 period (1998–2007, Javiana OR 1.23, Mbandaka OR 1.62; 2008–2018, Infantis OR 1.21; Appendix Table 3).

## Conclusions

*Salmonella* Reading was the serotype most strongly associated with illness during the Thanksgiving holiday. Given the dramatic increase in turkey consumption around Thanksgiving, one might expect that serotypes we identified are primarily associated with turkey consumption, and indeed, Reading caused a multistate outbreak with a raw turkey source during 2017–2019 (*8*), and a new clone of this serotype has emerged since 2014 in commercial turkey production (*9*). Other serotypes significantly associated with Thanksgiving in our study (i.e., Hadar, Schwarzengrund, and Heidelberg) have also been associated with turkey (*6*,*10*).

Other significantly associated serotypes are not among those most commonly identified in turkey (e.g., Heidelberg and 4,[5],12:i- are more commonly identified in chicken; Derby, Brandenburg, and 4,[5],12:i- in swine and pork; and 4,[5],12:i- in cattle). However, all these serotypes have been found in turkeys and in retail samples of turkey or have been associated with outbreaks attributed to turkey (*11*–*15*). Some of the serotypes significantly increased after Thanksgiving, such as, Baildon and Ohio, were rare, causing <200 illnesses annually, and were not reported among food animals, retail products, or outbreaks during 2015‒2020 (*7*). Although our study may have identified serotypes associated with other foods eaten during the Thanksgiving holiday, particular attention probably should be paid to evidence of these serotypes emerging in turkey production.

The first limitation of our study is that LEDS is a passive surveillance system and does not capture mild or asymptomatic infections for which ill persons do not seek healthcare or submit a specimen. Although we removed cases reported as outbreak-associated, those data are not reported by all states, and some outbreak-associated cases most likely are included. Missing data in LEDS varies, but serotype and date are largely complete. Although sensitivity analyses demonstrate consistency across time windows, misalignment of windows with causative exposures could have resulted in biases, possibly differential, from, for example, differences in healthcare seeking because of the holiday itself. Our study may be subject to ecologic bias because individual food exposures are unknown. We did not adjust for multiple testing because this analysis is intended to be hypothesis-generating rather than confirmatory.

Our case-crossover approach could be helpful for other pathogens and their subtypes that are likely to cause illnesses from a certain food disproportionately eaten during a brief period, such as turkey during the Thanksgiving holiday. Our technique provides unique insights into the causes of sporadic illness throughout the year and their changes over multiyear periods using no more than routine surveillance data and may provide valuable information to industry, regulators, and public health officials that could help guide monitoring and interventions to prevent illnesses and their associated morbidity and mortality. Consumers can also help protect themselves from *Salmonella* by following the 4 steps to food safety (https://www.cdc.gov/foodsafety/keep-food-safe.html).

Salmonella serotypes associated with illnesses after Thanksgiving.
