# Supplementary material for: Salmonella Serotypes Associated with Illnesses after Thanksgiving Holiday, United States, 1998–2018
Source: Emerg Infect Dis. 2022 Jan;28(1):210–3. doi: 10.3201/eid2801.211986 (PMC8714195; doi:10.3201/eid2801.211986)
Supplement: Supplementary file 1 — Salmonella serotypes associated with illnesses after Thanksgiving. [file 21-1986-Techapp-s1.pdf]

Article DOI: https://doi.org/10.3201/eid2801.211986

Salmonella Serotypes Associated with Illnesses after Thanksgiving Holiday, United States, 1998–2018

Appendix

Appendix Table 1. Demographic and clinical characteristics of laboratory-confirmed *Salmonella* infections (n=846,449) reported to the Laboratory-based Enteric Disease Surveillance system, United States, 1998–2018

Characteristic	No. cases	%
Total	846,449	100
Testing year		
1998–000	98,426	11.6
2001–2005	173,981	20.6
2006–2010	207,000	24.5
2011–2015	224,172	26.5
2016–2020*	142,870	16.9
Age (years)		
<5	204,997	25.9
5–9	63,411	8.0
10–19	75,929	9.6
20–29	83,702	10.6
30–39	73,582	9.3
40–49	74,437	9.4
50–59	75,716	9.6
60–69	62,786	7.9
>70	76,590	9.7
Missing	55,299	
Sex		
F	415,221	52.5
M	375,641	47.5
Missing	55,587	
Ethnicity		
Hispanic	18,565	12.4
Non-Hispanic	131,657	87.6
Missing	696,227	
Race		
White	200,429	82.2
Black/African-American	32,430	13.3
Asian/Pacific Islander	8112	3.3
American Indian/Alaska Native	2796	1.1
Missing	602,682	
Diagnosis type		
Culture	823,793	99.8
Culture-independent diagnostic testing	1919	0.2
Missing	20,737	
Specimen source		
Stool	675,017	86.8
Urine	49,205	6.3
Blood	48,675	6.3
Other	4614	0.6
Missing	68,938	
Salmonella serotype	No. cases	%
Enteritidis	142,687	18.3
Typhimurium	131,216	16.8
Newport	82,155	10.5
Javiana	41,144	5.3
Heidelberg	29,098	3.7
4,[5],12:i-	26,044	3.3
Infantis	17,866	2.3

Characteristic	No. cases	%
Hadar	5,967	0.8
Schwarzengrund	4,301	0.6
Mbandaka	4,246	0.5
Derby	2,579	0.3
Brandenburg	1,931	0.2
Reading	1,852	0.2
Ohio	1,360	0.2
Baildon	699	0.1
Worthington	650	0.1
Other serotype	286,617	36.7
Missing	66,037	
Outbreak-associated		
No	48,788	94.6
Yes	2805	5.4
Missing	794,856	

Appendix Table 2. Varying incubation, illness, and washout periods for laboratory-confirmed *Salmonella* infections with serotypes containing >50 cases during the study period

			Hadar		Reading		4,[5],12:i-		Derby		Heidelberg		Schwarzengrund	
Incubation, d	Illness, d	Washout, d	OR	p value	OR	p value	OR	p value	OR	p value	OR	p -value	OR	p value
12	7	5	1.02	0.853	1.87	<0.0001	1.07	0.258	1.18	0.261	1.08	0.142	1.36	0.022
12	5	5	1.06	0.688	1.97	<0.0001	1.07	0.334	1.2	0.324	1.14	0.037	1.8	<0.0001
12	5	2	1.08	0.625	1.99	<0.0001	0.97	0.617	0.97	0.872	1.26	0.001	1.73	0.001
12	5	0	1.11	0.487	2.14	<0.0001	1	0.955	1.02	0.932	1.29	<0.0001	1.75	0.001
10	7	5	1.11	0.411	1.79	<0.0001	1.09	0.156	1.29	0.097	1.21	0.001	1.63	0.001
10	5	5	1.08	0.576	2.44	<0.0001	1.12	0.91	1.41	0.039	1.34	<0.0001	1.72	0.001
10	5	2	1.34	0.055	2.25	<0.0001	1.01	0.924	1.26	0.1268	1.49	<0.0001	1.48	0.016
10	5	0	1.11	0.472	2.29	<0.0001	1.08	0.233	1.34	0.083	1.45	<0.0001	1.49	0.014
8	7	5	1.33	0.023	2.23	<0.0001	1.21	0.001	1.43	0.014	1.38	<0.0001	1.74	<0.0001
8	5	5	1.51	0.003	2.62	<0.0001	1.31	<0.0001	1.75	0.001	1.46	<0.0001	1.56	0.009
8	5	2	1.41	0.014	2.01	<0.0001	1.21	0.003	1.43	0.032	1.59	<0.0001	1.24	0.191
8	5	0	1.21	0.167	2.1	<0.0001	1.22	0.002	1.33	0.082	1.52	<0.0001	1.23	0.209
6	7	5	1.61	<0.0001	2.42	<0.0001	1.39	<0.0001	1.75	<0.0001	1.46	<0.0001	1.52	0.004
6	5	5	1.59	<0.0001	2.13	<0.0001	1.49	<0.0001	1.82	<0.0001	1.48	<0.0001	1.22	0.243
6	5	2	1.64	<0.0001	1.98	<0.0001	1.3	<0.0001	1.51	0.007	1.53	<0.0001	1.07	0.688
6	5	0	1.47	0.002	1.75	0.003	1.32	<0.0001	1.46	0.013	1.43	<0.0001	1.12	0.513
4	7	5	1.54	<0.0001	2.23	<0.0001	1.42	<0.0001	1.81	<0.0001	1.45	<0.0001	1.32	0.054
4	5	5	1.65	<0.0001	2.4	<0.0001	1.44	<0.0001	1.61	0.001	1.38	<0.0001	1.36	0.047
4	5	2	1.74	<0.0001	1.59	0.006	1.32	<0.0001	1.43	0.015	1.47	<0.0001	1.16	0.3336
4	5	0	1.58	<0.0001	1.22	0.223	1.32	<0.0001	1.41	0.021	1.34	<0.0001	1.01	0.965
2	7	5	1.66	<0.0001	2.18	<0.0001	1.39	<0.0001	1.57	0.001	1.34	<0.0001	1.4	0.018
2	5	5	1.78	<0.0001	1.85	<0.0001	1.36	<0.0001	1.51	0.008	1.26	<0.0001	1.22	0.193
2	5	2	1.76	<0.0001	1.31	0.085	1.21	0.001	1.28	0.097	1.2	0.002	1	0.99
2	5	0	1.6	<0.0001	1.32	0.082	1.15	0.015	1.18	0.269	1.08	0.199	1.05	0.728
1	7	5	1.72	<0.0001	1.79	<0.0001	1.34	<0.0001	1.48	0.005	1.23	<0.0001	1.27	0.088
0	7	5	1.77	<0.0001	1.4	0.023	1.31	<0.0001	1.41	0.015	1.18	0.003	1.09	0.549

*OR, odds ratio. Shading indicates statistical significance.

**Appendix Table 3.** ORs* for associated *Salmonella* serotypes during 1998–2007 and 2008–2018 and the entire evaluation period (1998–2018)Serotype	1998-2007			2008-2018			1998–2018		
	OR	95% CI	p-value	OR	95% CI	p value	OR	95% CI	p value
Reading	2.04	1.12–3.73	0.018	2.24	1.53–3.28	<0.0001	2.19	1.58–3.02	<0.0001
Baildon	1.08	0.18–6.44	0.936	2.06	1.07–3.95	0.026	1.92	1.05–3.53	0.032
Worthington	2.51	1.09–5.81	0.026	1.45	0.66–3.17	0.354	1.88	1.06–3.31	0.027
Ohio	1.33	0.65–2.7	0.429	2.29	1.13–4.64	0.018	1.75	1.06–2.86	0.025
Hadar	1.38	1.04–1.84	0.027	2.1	1.53–2.87	<0.0001	1.67	1.35–2.06	<0.0001
Derby	1.42	1–2.03	0.05	1.86	1.21–2.86	0.004	1.57	1.2–2.07	0.001
Brandenburg	1.34	0.85–2.11	0.207	1.57	0.92–2.67	0.095	1.42	1.01–2.01	0.044
Schwarzengrund	1.30	0.8–2.12	0.291	1.45	1.03–2.05	0.032	1.41	1.06–1.86	0.017
4,[5],12:i-	1.02	0.81–1.29	0.87	1.47	1.31–1.65	<0.0001	1.37	1.24–1.52	<0.0001
Heidelberg	1.36	1.18–1.55	<0.0001	1.36	1.15–1.59	<0.0001	1.35	1.21–1.49	<0.0001
Javiana	1.23	1.05–1.44	0.009	0.92	0.81–1.04	0.189	1.02	0.93–1.13	0.62
Mbandaka	1.62	1.06–2.46	0.023	0.75	0.5–1.12	0.157	1.03	0.78–1.36	0.82
Infantis	0.91	0.71–1.16	0.43	1.21	1.03–1.41	0.019	1.11	0.97–1.27	0.12

*OR, odds ratio. Shading indicates statistical significance.

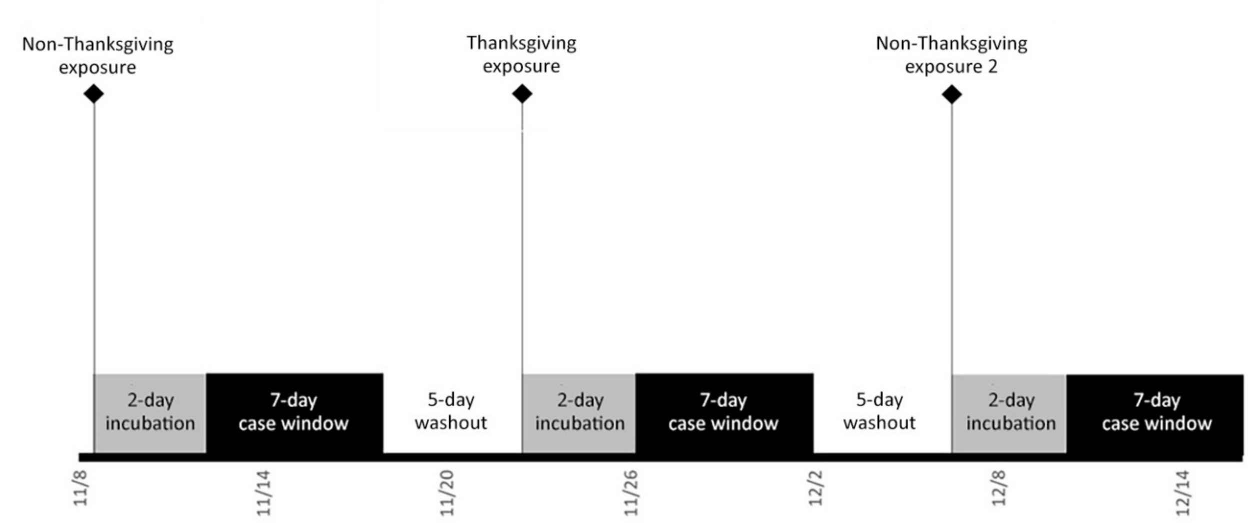

Appendix Figure. Example of case windows used in primary analysis before and after Thanksgiving Day, United States, 2017. *The 2-day incubation period represents the minimum incubation period; the 7-day case window includes the maximum incubation and reporting period.
